# Immune microenvironment in papillary thyroid carcinoma: roles of immune cells and checkpoints in disease progression and therapeutic implications

**DOI:** 10.3389/fimmu.2024.1438235

**Published:** 2024-09-03

**Authors:** Xun Zheng, Ruonan Sun, Tao Wei

**Affiliations:** ^1^ Department of Thyroid and Parathyroid Surgery, West China Hospital, Sichuan University, Chengdu, China; ^2^ West China School of Medicine, Sichuan University, Chengdu, China

**Keywords:** papillary thyroid cancer, immune microenvironment, immunization therapy, immune checkpoints, immune cells

## Abstract

Papillary thyroid cancer (PTC) is the most common type of primary thyroid cancer. Despite the low malignancy and relatively good prognosis, some PTC cases are highly aggressive and even develop refractory cancer in the thyroid. Growing evidence suggested that microenvironment in tumor affected PTC biological behavior due to different immune states. Different interconnected components in the immune system influence and participate in tumor invasion, and are closely related to PTC metastasis. Immune cells and molecules are widely distributed in PTC tissues. Their quantity and proportion vary with the host’s immune status, which suggests that immunotherapy may be a very promising therapeutic modality for PTC. In this paper, we review the role of immune cells and immune checkpoints in PTC immune microenvironment based on the characteristics of the PTC tumor microenvironment.

## 1 Introduction

Thyroid cancer (TC) represents a prevalent malignancy within the endocrine system, demonstrating a higher incidence in women compared to men and predominantly affecting individuals aged 40 to 50 (1, 2). The biological properties of various thyroid cancer subtypes span a broad spectrum. Based on their histological characteristics and cellular origins, thyroid cancers are classified into papillary, medullary, and follicular carcinomas (3). Papillary thyroid carcinoma (PTC) is a differentiated cancer subtype in the thyroid, constituting most form of primary thyroid malignancy (4). Over recent decades, the incidence of PTC has exhibited an increasing trend and a shift towards younger age groups (5, 6). For PTC, the current traditional therapies include surgical resection, radiotherapy, chemotherapy, endocrine inhibition and other therapeutic means, but the efficacy of various treatment methods has different degrees of limitations (7, 8). Despite their slow tumor growth, low malignancy, and overall favorable prognosis, over 10% of patients had tumor recurrence or metastasized to other sites after surgery (9). Some cases appear highly aggressive and may even progress to refractory thyroid cancer (10). Immune cell infiltration is frequently observed in the vicinity or within primary PTC tissue. The prognosis of PTC might be associated with the surrounding inflammatory response (11). Increasing evidence suggests that the immune microenvironment influences tumor biological behavior.

## 2 Immune cells In PTC tumor microenvironment

In 2002, Dunn proposed the immune editing hypothesis, which categorized the reciprocity between the tumors and immune system into “elimination”, “equilibrium”, and “escape”. The “elimination” phase, also called “surveillance”, involves the immune system clearing tumor cells before diagnosis. During the “equilibrium” phase, Tumor cells vary in the direction of low immunogenicity, which makes themselves not easily detected by the body’s immune surveillance mechanism (12). Studies (13, 14) have shown that tumor cells can “camouflage” themselves by reducing MHC I expression, thus evading immune system surveillance. Another study (15) analyzed the influence of the immune environment on the clinical manifestations of patients and found that immune cells in PTC patients’ thyroids differed from healthy ones. Specifically, the proportions of B cells, T cells (mainly CD8+ T cells) and M1 macrophages showed obvious reduction. The larger the difference between these immune cells and healthy thyroid tissue, the greater the likelihood of PTC progression and recurrence, and the lower the patients’ overall survival rate.

The tumor microenvironment (TME) contains tumor cells and their living environment (including immune cells, stromal cells and blood vessels), which cooperate with each other (16). Each component in TME plays a crucial role in tumor initiation and progression. Their quantity as well as proportion vary with the host’s immune status (17). In most cancers, a high proportion of M2/M1 macrophages is strongly associated with poor clinical prognosis (18). In thyroid cancer, tumor-related macrophages (Tumor-associated macrophages, TAMs) are dominated by M2 polarized macrophages, providing a good tumor microenvironment for tumor growth, survival and angiogenesis. Experimental results of various tumors, including thyroid cancer, show that high-density TAMs are associated with poor prognosis of tumors (19, 20). At present, many cytokines, chemokines and their signaling pathways also have been found in PTC. For example, activation of IL-6/JAK2/STAT3 pathway could promote PTC cell proliferation and migration, and IL-34 promotes PTC cell proliferation (21), epithelial-stromal transition and extracellular regulatory kinase signaling pathway and inhibits apoptosis (22). In PTC tumor microenvironment, overexpression of IL-6 promotes the growth of PTC (23). The infiltration of plasma cells in the DTC microenvironment was positively correlated with a favorable prognosis (24). Immature Dendritic cells in the PTC microenvironment can secrete immunosuppressive cytokines, such as IL-10 and TGF- β, so as to inhibit the immune response and result in the development of PTC, while CD8^+^ T cells recognize tumor cells to express antigen and thus participate in the killing of tumor cells, exhibiting protective effects on PTC (25, 26).

Xie Z et al. (27) investigated immune-related cells in TME, focusing on the relationship between PTC and chronic inflammation. The study included 799 PTC patients and 194 healthy ones. It was found that compared with normal thyroids, the overall immune level of PTC tissues was stronger, and many cells in TME such as Tregs and M0 macrophages were elevated. Furthermore, the more advanced the tumor, the greater the proportion and abundance above normal levels. Higher immune group had a later stage than the lower one, with a larger tumor size, increased metastasis of lymph node, and a higher frequency of BRAF mutations. This suggests that changes in immune status within the TME are closely related to tumor progression, and that various immune cells can either promote or inhibit PTC metastasis and recurrence to different extents.

### 2.1 Natural killer cells

NK cells are essential components of inherent immunity that express various regulatory receptors associated with activation or inhibition. These receptors facilitate the distinction between “self” and “non-self,” enabling them to selectively “eliminate” (28). NK cell infiltration in tumors is often linked to the initiation or progression of cancer of early and metastatic stages of tumor development, and is generally predictive of a favorable prognosis (29).

In PTC, NK cells in TME are elevated in comparison to normal thyroid tissue, but not in peripheral blood (30). The abundance of NK cells in TME is significantly negatively associated with tumor progression. NK cells are able to kill cancer cells directly, and also responsible for the immune surveillance (31, 32). They may provide new ideas for PTC diagnosis and therapy. However, their efficacy is somewhat limited during the anti-tumor process due to the secretion of immunosuppressive factors by tumor cells, which reduce the activation receptors on NK cells while upregulating inhibitory receptors, making NK cell activation difficult. Tumor cells can also evade immune surveillance by reducing MHC I molecule expression, which blocks tumor antigen presentation (33). Additionally, the number and functionality of NK cells in the TME typically decline with tumor progression (34), and NK cells may be rendered dysfunctional due to metabolic disorders (35). These limitations of NK cells within the TME should be considered when utilizing them for PTC diagnosis, staging, and treatment.

### 2.2 T lymphocytes

T lymphocytes can be classified into helper T cells (Th), cytotoxic T cells (CTL), and regulatory T cells (Treg) according to their various functions. They mature from lymphoid progenitor cells in the thymus and are central to cellular immunity. CD4 is expressed in all Th cells. Naive CD4+T cells, known as Th0 cells, can differentiate into Th1, Th2, and Th17 lineages that have distinct immune roles through antigen stimulation and cytokine regulation. Th1 cells enhance and amplify cellular responses by secreting regulatory molecule, including interleukin (IL)-2 and IFN-γ, and induce other immune cells to exhibit antitumor activity (36). In contrast, Th2 cells inhibit the antitumor effects of cellular immunity by secreting IL-4 and suppressing NK cell activation (37). The Th1/Th2 ratio serves as a useful indicator of dynamic changes in the antitumor immune process. Moreover, Th17 levels in PTC tissue samples are higher than in healthy thyroid tissue, with this difference also observed in patients’ peripheral blood. More Th17 in peripheral blood tend to predict larger tumor volume (38).

The primary function of CTLs is to specifically recognize endogenous antigen peptide-MHC I molecular complexes and subsequently kill tumor cells. This has become an essential marker for evaluating tumor prognosis (39–41). PTC patients with a higher expression of CD8+ CTLs show lower tumor stages and higher survival rates, while the reduction of CD8+ T cells weakens the immune system’s ability to eliminate tumor cells, making tumors more aggressive (42). In the study by Modi J et al (43). PTC patients with CD8+ T cell infiltration experienced slower tumor progression, reduced tumor growth, and fewer recurrences.

Tregs, commonly referred to as CD4+CD25+Foxp3+ T cells, primarily weaken immune level through direct contact to target cells and cytokine secretion. High Tregs expression in cancer tissue is typically related to poor prognosis. Tregs are highly aggregated in the tumor site and peripheral blood of cancer patients (44, 45), and their inhibitory effect on the immune function of cancer patients is stronger than in healthy individuals (46). Tregs in PTC patients’ peripheral blood are significantly increased compared to normal thyroid tissue and thyroid adenoma patients (47, 48). In the TME, Tregs can weaken the body’s immune response to tumors through various mechanisms, including affecting cytokine secretion (49, 50), increasing cAMP-mediated immunosuppression via adenosine and prostaglandin (51, 52), regulating signal transduction through receptor-ligand binding (53, 54), and mediating immunosuppression through the exosome pathway (55). French JD et al. (42) using immunohistochemical analysis, quantitatively counted lymphocytes in the TME of PTC tissues, and found that T cells in the PTC tissues of patients were mainly composed of CD4 + T cells. The quantity of Foxp3+ regulatory T cells was related to lymph node metastasis (r = 0.858; P = 0.002), and the ratio of CD8 to Treg was strongly negatively associated with tumor size.

In the future, the frequency of Treg cells in TME is likely to become an important factor in predicting, diagnosing, and evaluating the prognosis of PTC. Furthermore, the suppressive effect of Treg cells should be taken into account when designing immunotherapy for PTC. Overall, a better understanding of the complex interactions between various immune cell types in the TME is significant for the exploration of more effective diagnostic and therapeutic strategies for PTC and other cancers.

### 2.3 Mast cells

Mast cells are tissue-resident component ubiquitously distributed across nearly all tissues. Their regulatory role in the tumor microenvironment (TME) is often multifaceted, exhibiting both pro-tumorigenic and anti-tumorigenic effects (56). The tumor-promoting effects primarily involve the secretion of vascular endothelial growth factors (VEGF) to promote neovascularization, the secretion of matrix metalloproteinases (MMPs) to enhance cancer progression, and the release of regulatory molecules to facilitate immune tolerance. Conversely, their anticancer effects include direct inhibition of tumor growth, immune stimulation, and reduction of cell motility (57). Mast cells situated within or surrounding tumors may exhibit different roles. While mast cells generally play a pro-carcinogenic role in most tumors (58, 59), their contributions to cancer progression can vary depending on which stage the tumors are at and where they are in tumor tissue (60).

Limited studies (61) have assessed the correlation between mast cells and PTC. One study reported that mast cell accumulation was observed in 95% of PTC samples, with the density positively correlated with cancer aggressiveness. Other studies demonstrated that mast cell derivatives, such as histamine and chemokines, accelerated the progression of PTC as well as distant metastasis *in vitro*. But this phenomenon will be exactly the opposite when inhibitors of mast cells are applied (62), potentially providing novel therapeutic strategies for PTC treatment.

### 2.4 Tumor-associated macrophages

Tumor-associated macrophages (TAMs) are the most abundant in the tumor microenvironment (TME). They can differentiate into two subpopulations that exert opposing effects on the host’s immune response to tumors. M1 macrophages predominantly suppress tumor growth and angiogenesis by producing cytokines like IL-1. In contrast, M2 macrophages generate IL-13, IL-10, and other factors that foster tumor development and enhance the invasive capabilities of tumor cells (63). Within the TME, cancer cells secrete signaling factors, mediated by exosomes, that induce mononuclear macrophages to differentiate into the M2 subtype (64), resulting in an imbalance between M1 and M2 populations and ultimately promoting cancer progression (65).

Elevated TAM in PTC is closely related with biological behavior of the tumors (66). Studies (67, 68) have revealed the macrophage infiltration rate in PTC is significantly higher than that in benign tumors, with the extent of infiltration positively correlating with lymph node metastasis. The underlying mechanism remains incompletely understood; however, it may involve TAMs promoting tumor cells of PTC metastasis through the cytokine CXCL8 and its paracrine interaction with CXCR1/2 (69). Consequently, a comprehensive understanding of the functional differences between distinct TAM subtypes in the thyroid gland may potentially establish TAMs as new idea for thyroid tumor therapy.

### 2.5 Dendritic cells

Dendritic cells (DCs) are the most functionally specialized APCs in immune system. They serve as initiators of the adaptive immune response and act as a “bridge” connecting innate and adaptive immunity.

Normally, DCs are scarcely present in thyroid tissue. However, their prevalence increases in human papillary thyroid carcinoma (PTC) tissue (70). Immature DCs possess robust antigen-processing capabilities but are less effective in promoting immune responses. Interestingly, they may even weaken immune responses by secreting inhibitory cytokines including IL-10 and TGF-β (71).

Moreover, Tregs and DCs can interact and collaboratively involve in immune regulation in TME. In PTC tissues, Tregs can inhibit DC function, co-stimulatory ligands expression, CD8+ T cells activation (72). DCs are able to restore their function by blocking PD-1 pathways, IL-10 secretion, and production of lactic acid (73). Therefore, disrupting the interaction between Tregs and DCs in PTC may shed new light on immune therapy.

### 2.6 Neutrophils

Neutrophils have long been recognized for their pivotal role in acute phase of inflammatory. Recently, they’ve emerged as a new subject of investigation in the field of oncology. Accumulating experimental evidence suggests that neutrophils may exert both antitumor and protumor effects by releasing various regulatory molecules within the tumor microenvironment (74). Neutrophils exhibit a dual role in PTC development and progression. On one hand, they promote genetic instability, proliferation, invasion (75), and vascular remodeling of cancer cells by releasing neutrophil elastase (76). Conversely, neutrophils have demonstrated antitumor properties, possessing the capacity to “eliminate” through antibody-dependent cellular cytotoxicity (ADCC) (77). Maria et al. found that PTC tissue extended the survival of human neutrophils and enhances its activity and reactive oxygen species (ROS) generation, suggesting that neutrophils can acquire a cytotoxic antitumor phenotype under the influence of thyroid tumor microenvironment. Notably, during tumor progression, the neutrophil population increases, and their phenotype undergoes alterations. Several subsets of circulating neutrophils with distinct maturity and immunological properties can be identified in advanced cancer, each playing a unique role in tumor immunity (78).

In PTC tissues, tumor cells recruit neutrophils by releasing CXCL8/IL-8 and reduce apoptosis rate of neutrophils through secretion of granulocyte colony-stimulating factor (GM-CSF) (79). The ratio of neutrophil count to lymphocyte count (neutrophil to lymphocyte ratio; NLR) in peripheral blood is associated with tumor development and progression (80), and higher NLR is associated with larger tumor volume and higher risk of recurrence in thyroid cancer (81).

## 3 Immune checkpoints of PTC

Lymphocyte activation primarily relies on the specific recognition of antigens by antigen receptors, with the strength, duration, and nature of the activation signal often regulated by cell surface receptor molecules. Immune checkpoints act as regulatory components, controlling timing and intensity of immune responses, maintaining self-tolerance, and preventing immune hyperactivity. In TME, these regulators inhibits immune responses, rendering the body incapable of mounting an efficient immune response against cancer, thus facilitating immune evasion (82). Common immune checkpoints in PTC include programmed cell death protein 1 (PD-1), programmed cell death ligand 1 (PD-L1), cytotoxic T lymphocyte antigen 4 (CTLA-4), and indoleamine 2,3-dioxygenase (IDO) (83).

A recent study (15) revealed that several key immune checkpoints, including LAG3, PD-1, and IDO1, are inhibited in early PTC compared to normal thyroid tissue, potentially associated to the prevention of immune cell-mediated damage to healthy thyroid tissue. Interestingly, during the pathological stage, most of the immune checkpoints were upregulated, particularly the N stage, advanced. Likewise, the BRAFV600E mutation has been associated with the elevation of most checkpoints (84, 85).

### 3.1 Programmed cell death protein 1/Programmed cell death ligand 1

The PD-1/PD-L1 pathway has emerged as a vital suppressive regulator in cancer. The overexpression of PD-L1 suggests that PD-L1 undermines immune surveillance of tumor in TME (86). Due to the cell and tissue-specific distribution of PD-L1, PD-1 play its part in at distinct stages of T cell activation, altering T cell function under antigen-specific stimulation, inhibiting CTLs, and enhancing tumor proliferation and invasion (87, 88). When T cells are recognized with PD-L1-positive tumor cells, tumor cells can cause programmed T cell death. In addition, tumor cells can produce cytokines including IL-10, allowing tumor cells to escape the clearance of CTL (47).. These mechanisms facilitate immune evasion by thyroid cancer cells and play a critical role in the transformation of normal cells into tumor cells (89).

PD-1 is widely expressed on lymphocytes capable of receiving antigen stimulation, acting as a “rheostat” for immune responses and regulating lymphocyte reactions to antigens. During antigen recognition, PD-1 binds to its ligands, recruiting tyrosine phosphatase (SHP-2), which can dephosphorylate and inactivate proximal effector molecules of antigen receptors on lymphocyte surfaces (87), such as inactivating Zap70 in T lymphocytes to inhibit TCR signaling (90) or inactivating Syk in B lymphocytes to inhibit BCR signaling (91).

**Figure 1:**
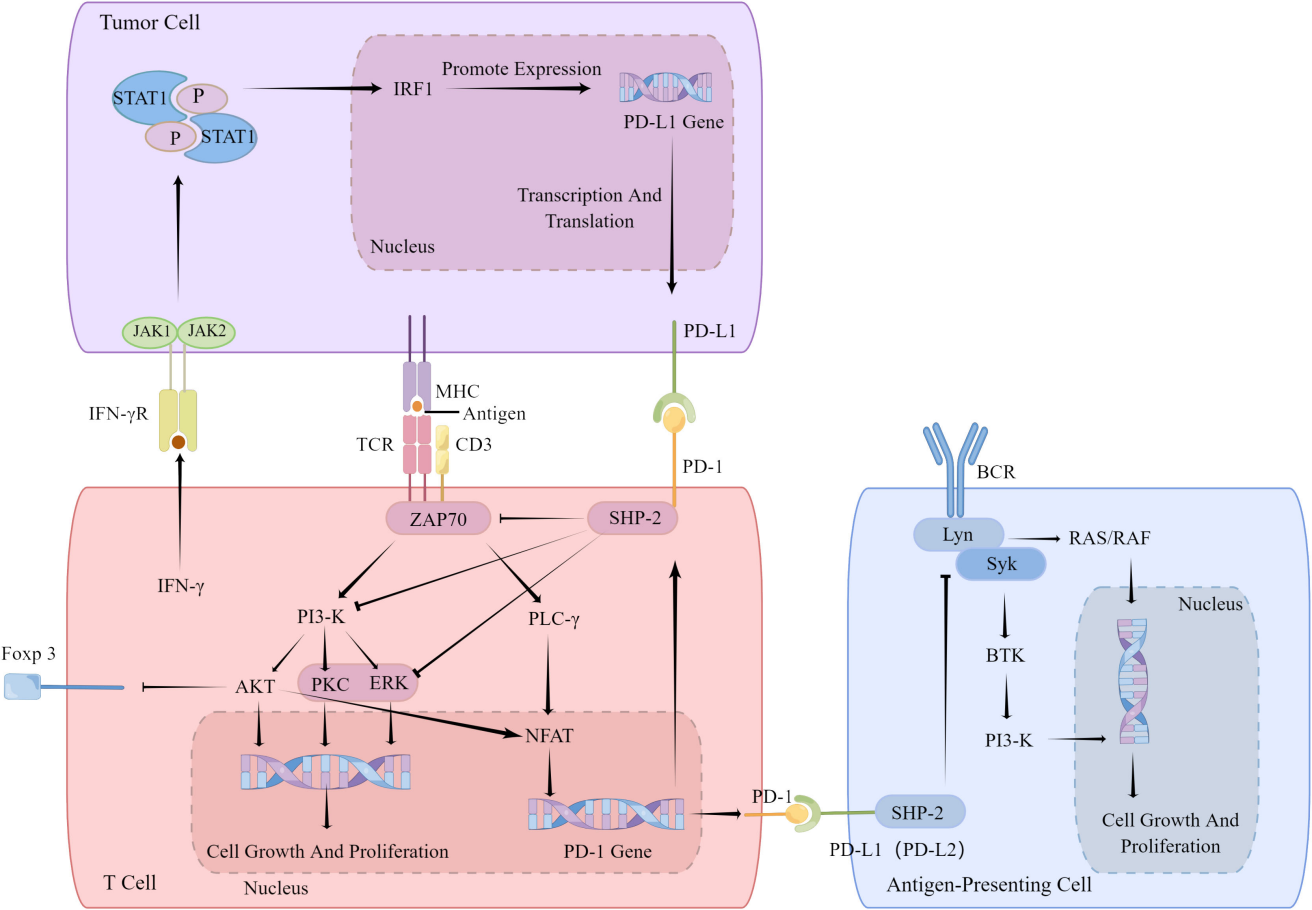
PD-1 inhibit TCR and BCR signaling. PD-1 inhibits the co-stimulatory signal of T cell activation by raising SHP-2,so that T cells cannot be activated normally and lead to increases Foxp3 expression. IFN-γ secreted by T cells will induce tumor cells to express PD-1 receptor PD-L1. PD-1 inhibits B cell activation by inhibiting downstream signal of BCR. IFN-γ, interferon-γ; IRF1, Interferon regulatory factor 1; CD3, coreceptor; PI3-K, SHP-2, ZAP70, JAK1 and JAK2, kinases; PLC-γ, phospholipase C-γ; AKT, kinase; PKC, Protein kinase C; ERK, extracellular regulated protein kinases; NFAT, activating T nuclear factor; NF-κB, transcription factor; Lyn, Syk, BTK, kinases.

PD-1’s effects on biochemical signaling pathways also promote T cell conversion of naive into inducible Treg (iTreg) cell through various mechanisms. Firstly, PD-1 enhances Foxp3 expression by inhibiting Akt activation (92). Secondly, by inhibiting cyclin-dependent kinase 2 (Cdk2), PD-1 amplifies Smad3-mediated transactivation by transforming growth factor β (TGF-β) (93, 94), promoting Foxp3 transcription (95). Thirdly, through metabolic reprogramming of activated T cells, PD-1 inhibits glucose metabolism (96) and promotes fatty acid β-oxidation (97), specifically activating metabolic programs that support Treg cell generation while inhibiting Th0 cell differentiation into Th1 or Th17 cells (98, 99). Therefore, targeting PD-1 and its downstream signaling pathways is an effective means of improving immunity in cancers. The PD-1 pathway represents one of the primary factor in immune escape. Given their specificity and significance, PD-1-blocking agents have shown considerable promise in cancer immunotherapy. Currently, these agents are widely employed in diagnosing and treating clinical diseases, exhibiting high clinical value for advanced cancers. They hold the potential to control other immune diseases through PD-1 signaling as well (100) (**Figure 1**).

### 3.2 Cytotoxic T lymphocyte antigen-4

**Figure 2:**
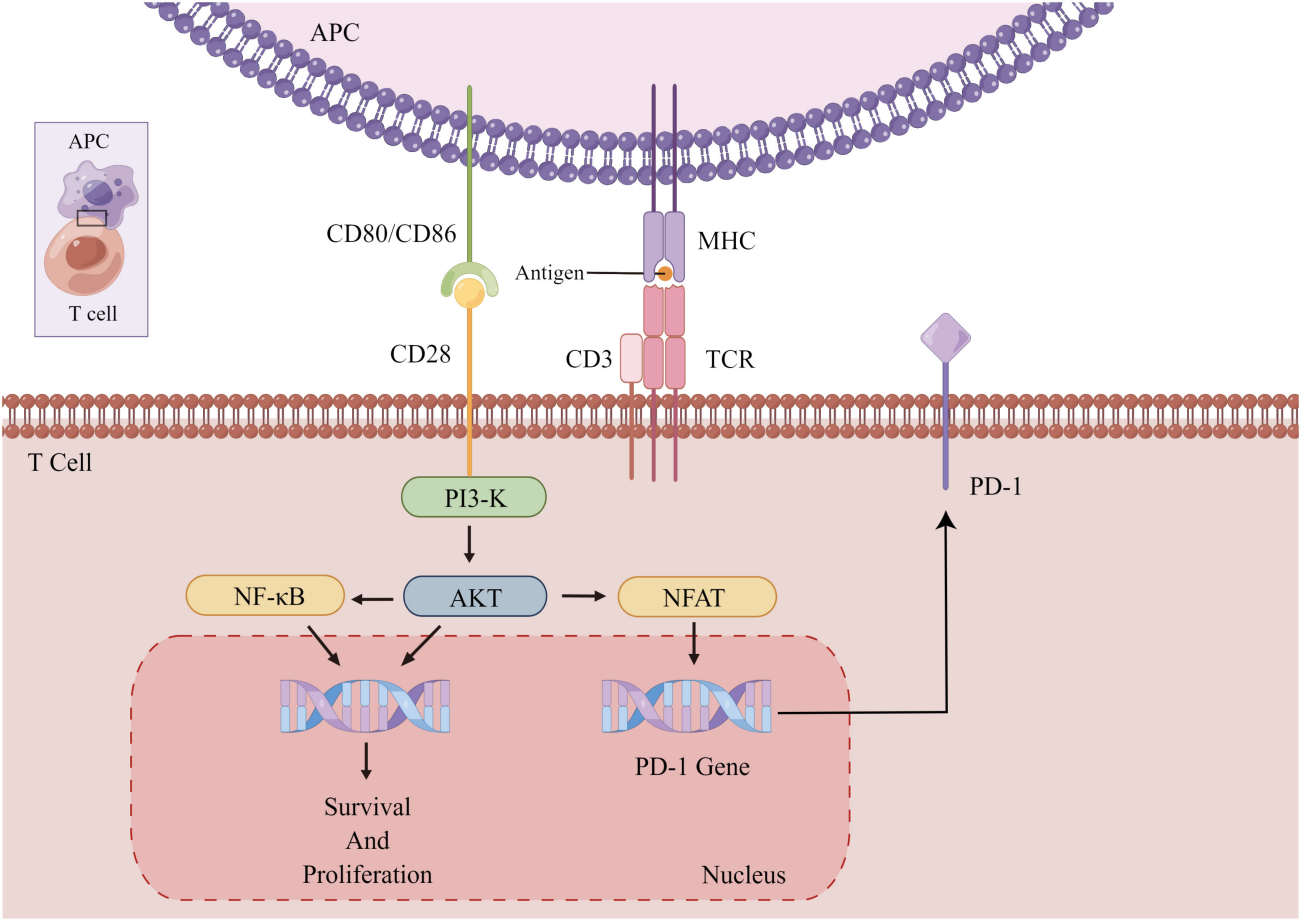
Activation and proliferation of normal T cells. The binding of CD28 and CD80/86 provides a co-stimulatory signal for T cell activation, causing T cell activation and proliferation. Chronically activated T cells increase the expression of PD-1 to prevent immune overshoot. APC, antigen-presenting cells; PI3-K, AKT, kinase; NF-κB, transcription factor; CD3, coreceptor; NFAT, activating T nuclear factor.

**Figure 3:**
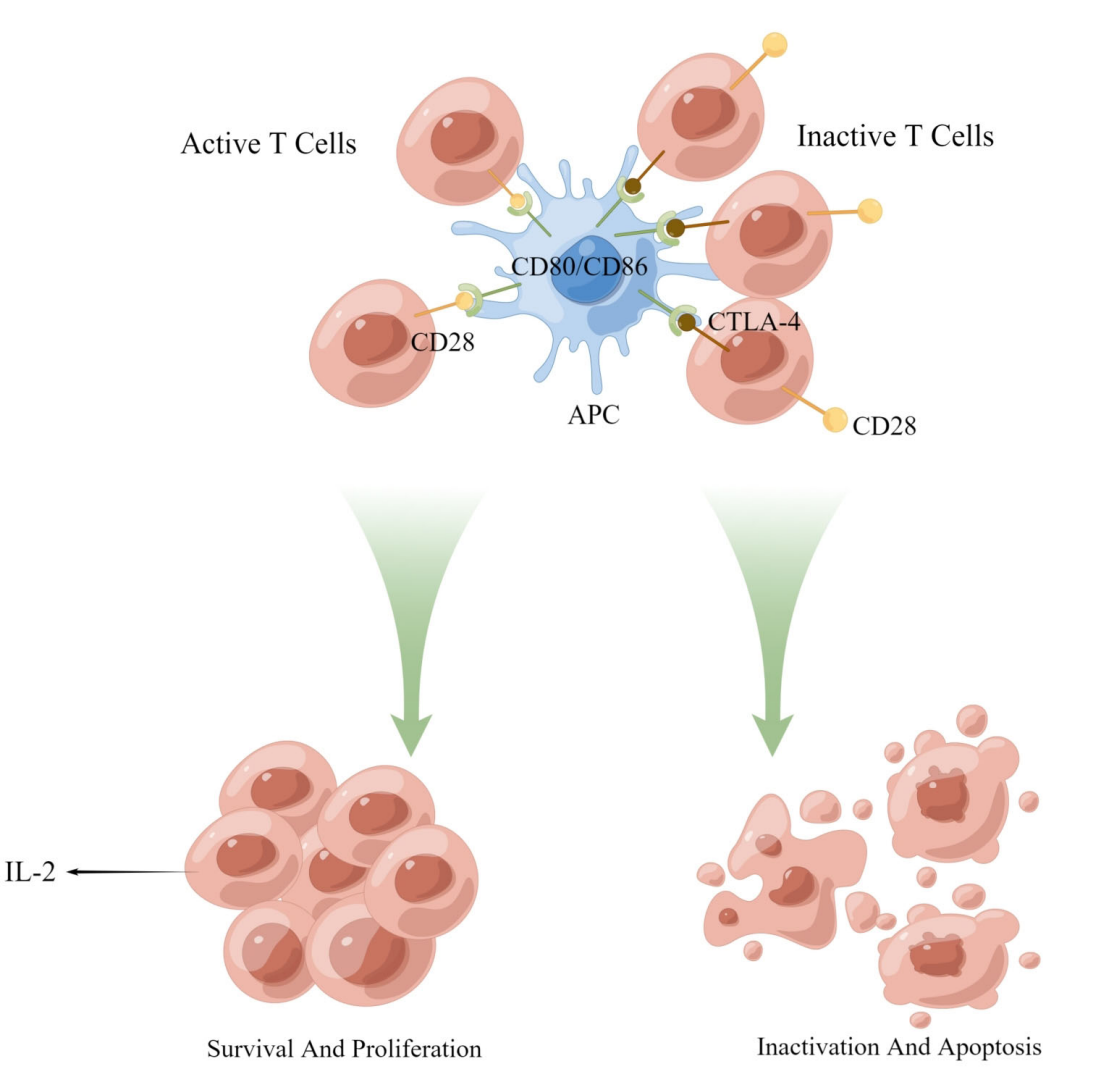
Activation and proliferation of normal T cells. The binding of CD28 and CD80/86 provides a co-stimulatory signal for T cell activation, causing T cell activation and proliferation. Chronically activated T cells increase the expression of PD-1 to prevent immune overshoot. APC, antigen-presenting cells; PI3-K, AKT, kinase; NF-κB, transcription factor; CD3, coreceptor; NFAT, activating T nuclear factor.

CTLA-4 is a transmembrane protein implicated in immune regulation, typically occur on activated T cells. It attenuates T cell activation primarily by inhibiting the CD28 costimulatory signal (**Figure 2**). This is partially due to its competition with CD28 for recognition to CD80 and CD86 on APCs, which obstructs costimulatory signals essential for T cell activation and prevents downstream signal transduction promoting T cell activation and proliferation (101, 102). Consequently, CTLA-4 makes it difficult for T cells to activate. Upon CTLA-4 activation, T cell activation and IL-2 secretion are diminished, exerting a negative regulatory effect on tumor immunity (**Figure 3**). Recent studies have also demonstrated that PD-1+Tim-3+CD8+ T lymphocytes exhibit varying degrees of functional impairment in patients with regional metastatic PTC (103).

In comparison, PD-1 indirectly hinders TCR or BCR responses to antigens via intracellular signaling, while CTLA-4 entirely obstructs CD28 costimulation through competitive inhibition, acting more comprehensively and rapidly (87).

### 3.3 Indoleamine 2, 3-dioxygenase 1

Indoleamine 2,3-dioxygenase 1 (IDO1) is a oxidoreductase responsible for catalyzing. In papillary thyroid microcarcinoma (PTMC), 31% of the cells were positive for IDO, which may be associated with tumor metastasis (104). In cancer, IDO1 can exert an immunosuppressive function, and its expression is significantly correlated with FoxP3. This relationship promotes tumor immune evasion by inducing FoxP3 phenotype regulation, consequently suppressing the immune microenvironment (105).

## 4 Regulatory effect of BRAF V600E mutation

BRAF is an activator of the RAS-regulated serine-threonine kinase and the MAPK signaling cascade. This pathway mediates the regulation of cell proliferation, differentiation, and survival in response to extracellular signals. The BRAFV600E mutation simulates phosphorylation in the activating fragment of BRAF, resulting in the dysregulation of cell proliferation (106).

The BRAFV600E gene mutation is closely related to elevated quantity of immunosuppressive regulators in PTC cells. Studies have reported (24) that CTLA-4 and PD-L1 expression levels are inversely associated with thyroid differentiation score (TDS) in PTC, a relationship more pronounced in tumors harboring the BRAFV600E mutation. BRAFV600E tumors expressed higher levels of PD-1 compared to BRAF wild-type tumors (53% vs. 12.5%). BRAFV600E promotes thyroid cancer development by increasing myeloid-derived suppressor cells (MDSCs) (107). As a heterogeneous population of immature myeloid cells, MDSCs are the primary coordinator of the immunosuppressive environment in cancer. MDSCs, primarily through CXCR2, show ligand recruitment to the TME (108). MDSCs are amplified during cancer progression and has the remarkable ability to inhibit T cell function in the tumor microenvironment (109), which is able to produce mediators necessary for neoangiogenesis and tissue invasion (110). In the peripheral circulation, MDSCs promote PTC progression. By inhibiting miR-486-3p, MDSCs promoted the activity of the NF-κB2 signaling pathway, leading to the accelerated invasion (111).

In addition, BRAFV600E upregulated T-box transcription factor 3 (TBX3) induced MAPK pathway activation. Therefore, TBX3 could be associated with BRAFV600E-related tumor genesis (112). TBX3 belongs to the T-box transcription factors family, associated with tumor progression and metastasis (113). Analysis of PTC patient specimens revealed that TBX3 is highly expressed in cancerous thyroid cells, indicating down regulation of TBX3 could delay the G1/S phase transition, decreased cell growth *in vitro* and inhibited tumor formation *in vivo (* 114).

Considering the strong correlation between BRAF and the pathological characteristic of PTC, BRAF mutation status has the potential to serve as a risk assessment indicator and prognostic marker for PTC. However, similar prediction models are challenging to adapt to multivariate factors, such as patient age and gender, which may increase the cost and complexity of evaluation. These limitations necessitate further exploration (115). Beyond risk assessment and prognosis, the BRAF mutation may play a crucial role as a therapeutic target for PTC. Currently, BRAF kinase inhibitors have been utilized in non-small cell lung cancer and melanoma, while research on PTC treatment remains in its early stages (116).

## 5 Immunotherapy strategies for PTC

For patients with advanced PTC or distant metastases, conventional therapies, including chemotherapy and radiotherapy, are prone to developing tolerance (117), thereby limiting their effectiveness. Consequently, treatment options for patients with advanced disease or distant metastases are restricted. Harnessing the immune system appears to be a highly promising strategy for addressing these challenges.

### 5.1 Adoptive cell therapy

Adoptive cell therapy (ACT) involves the extraction of precursor cells from autologous or allogeneic anti-tumor effector cells, followed by their *in vitro* induction, activation, and expansion using activators such as IL-2 and specific peptides. Proliferating cells are then transfused back into cancer patients and enhance their anti-tumor immunity, aiming to achieve therapeutic effects and prevent recurrence (118, 119).

Phase I clinical trial results have demonstrated that dendritic cells stimulated with autologous PTC tumor lysates can effectively control tumor progression without significant adverse effects (120). In this study, patients with refractory PTC and distant metastases were selected, and some experienced stabilization after treatment, confirming the feasibility of ACT for advanced PTC management.

Apart from DCs, chimeric antigen receptor T (CAR-T) cell immunotherapy has also undergone modifications and been applied in clinical practice in recent years. Genetic engineering techniques enable the addition of chimeric antibodies to T cells, allowing T cells to recognize and simultaneously activate tumor cell killing. There has been preclinical validation on the therapy for intercellular adhesion molecule (ICAM)-1 in thyroid cancer. Based on previous study findings (121), some investigators (122) have verified the feasibility of ICAM-1 as a CAR-targeting antigen by examining its relationship with tumor malignancy in patients with recurrent advanced PTC lacking other treatment options. Other studies (123, 124) have also reported a favorable safety profile for this therapy, suggesting the potential of ICAM-1 as a target for treatment of advanced recurrent thyroid tumors.

Since T cells upregulate ICAM-1 expression upon activation, ICAM-1 CAR-T cells may engage in mutual attacks, potentially reducing T cell infiltration into PTC tissues and causing collateral tissue damage (125). Therefore, further refinement is necessary before this therapy can be widely adopted in clinical practice.

### 5.2 Immune checkpoint inhibitors

Immune checkpoint inhibitors (ICIs) are monoclonal antibody (mAb) drugs developed to target specific immune checkpoints. Tumor cells cannot interact with immune cells through immune checkpoints above when ICIs are applied, which can block immune checkpoint-mediated immune escape. There has been monoclonal antibodies against PD-1/PD-L1 and CTLA-4, such as pembrolizumab and ipilimumab (126).

Existing trials have demonstrated that ICIs exhibit good efficacy and safety in PTC treatment (126, 127). The potential of combining ICIs with currently available drugs for advanced thyroid cancer has garnered interest. Animal studies have confirmed that combinations of BRAF inhibitors and checkpoint inhibitor immunotherapies synergistically reduce tumor volume in mouse models of carcinoma (128). However, mAbs can sometimes cause immune-related adverse events resembling autoimmune reactions (129), prompting consideration of small molecule inhibitors as alternative therapeutic strategies. Unlike mAbs, small molecule inhibitors can interact with both receptor on the surface and intracellular molecular targets (26), making them a promising therapeutic approach.

The efficacy of ICIs is influenced by the host’s immune status, as they target immune checkpoints and the function of immune cells and molecules in TME changes accordingly. Intrinsic microorganisms contribute to the body’s overall and local immunological regulation and can significantly impact the efficacy of ICIs (130). In PTC, VEGF can inhibit DC antigen presentation, enhance Treg amplification, and mediate the upregulation of PD-1 on T cells in TME. Combining VEGF inhibitors with ICIs can synergistically promote immune checkpoint blockade effect (131–133). Given the unique influence of the immune microenvironment on tumor progression, the combination of anti-inflammatory drugs and ICIs is also common. For instance, aspirin is widely used in cancer treatment and can reduce the mortality rate of various adenocarcinomas (134). Metformin and phenformin affect angiogenesis (135), regulate immune responses (136), and can be used in combination with ICIs. Consequently, to widely apply ICIs in the clinical treatment of PTC, a comprehensive assessment of the patient’s immune status is necessary.

## 6 Conclusion

In summary, immune cells and molecules in TME are of vital importance in papillary thyroid carcinoma (PTC) progression by modulating immune response against cancer. Immune checkpoints are regulatory molecules in the immune system, with the PD-1/PD-L1 and CTLA-4 pathways emerging as significant contributors to tumor immunosuppression. Furthermore, the BRAFV600E mutation is intimately linked to PTC development and progression, potentially leading to aberrant cell proliferation and subsequent PTC onset. BRAFV600E also exerts a regulatory effect on immune checkpoints. CTLA-4 and PD-L1 levels are inversely associated with TDS, particularly in tumors harboring the BRAFV600E mutation. Consequently, BRAFV600E may serve as a critical target and prognostic marker for PTC treatment.

Patients with advanced disease or distant metastases face limited treatment options, making the utilization of the immune system a particularly promising approach. Adoptive cell therapy, utilizing dendritic cells (DC) and chimeric antigen receptor T (CAR-T) cells, has proven effective for patients with advanced PTC. Employing immune checkpoint inhibitors (ICIs) to modulate PD-1 targets and their downstream signaling pathways effectively enhances the host’s immunity to cancer; however, ICIs can sometimes result in immune-related adverse events, warranting consideration of small molecule inhibitors as an alternative. Moreover, ICI efficacy is easily influenced by gut microorganisms and the body’s immune levels, necessitating the assessment of the host’s immune status during treatment. Combination of ICIs with vascular endothelial growth factor (VEGF) inhibitors or anti-inflammatory drugs has demonstrated improved efficacy and is expected to offer potential therapeutic value for PTC management.
